# Identifiable Images of Bystanders Extracted from Corneal Reflections

**DOI:** 10.1371/journal.pone.0083325

**Published:** 2013-12-26

**Authors:** Rob Jenkins, Christie Kerr

**Affiliations:** 1 Department of Psychology, University of York, York, North Yorkshire, United Kingdom; 2 School of Psychology, University of Glasgow, Glasgow, Lanarkshire, United Kingdom; Birkbeck, University of London, United Kingdom

## Abstract

Criminal investigations often use photographic evidence to identify suspects. Here we combined robust face perception and high-resolution photography to mine face photographs for hidden information. By zooming in on high-resolution face photographs, we were able to recover images of unseen bystanders from reflections in the subjects' eyes. To establish whether these bystanders could be identified from the reflection images, we presented them as stimuli in a face matching task (Experiment 1). Accuracy in the face matching task was well above chance (50%), despite the unpromising source of the stimuli. Participants who were *unfamiliar* with the bystanders' faces (n = 16) performed at 71% accuracy [*t*(15) = 7.64, *p*<.0001, *d* = 1.91], and participants who were *familiar* with the faces (n = 16) performed at 84% accuracy [*t*(15) = 11.15, *p*<.0001, *d* = 2.79]. In a test of spontaneous recognition (Experiment 2), observers could reliably name a familiar face from an eye reflection image. For crimes in which the victims are photographed (e.g., hostage taking, child sex abuse), reflections in the eyes of the photographic subject could help to identify perpetrators.

## Introduction

Cameras are routinely seized as evidence during criminal investigations [1]. Images of people retrieved from these cameras may be used to piece together networks of associates, or to link individuals to particular locations. In particular, it may be desirable to identify the photographer, or other individuals who were present at the scene but were not directly captured in the photograph. Bystander identification may be especially important when the images record criminal activity, as when hostage takers or child sex abusers photograph their victims [2] [3].

**Figure 1:**
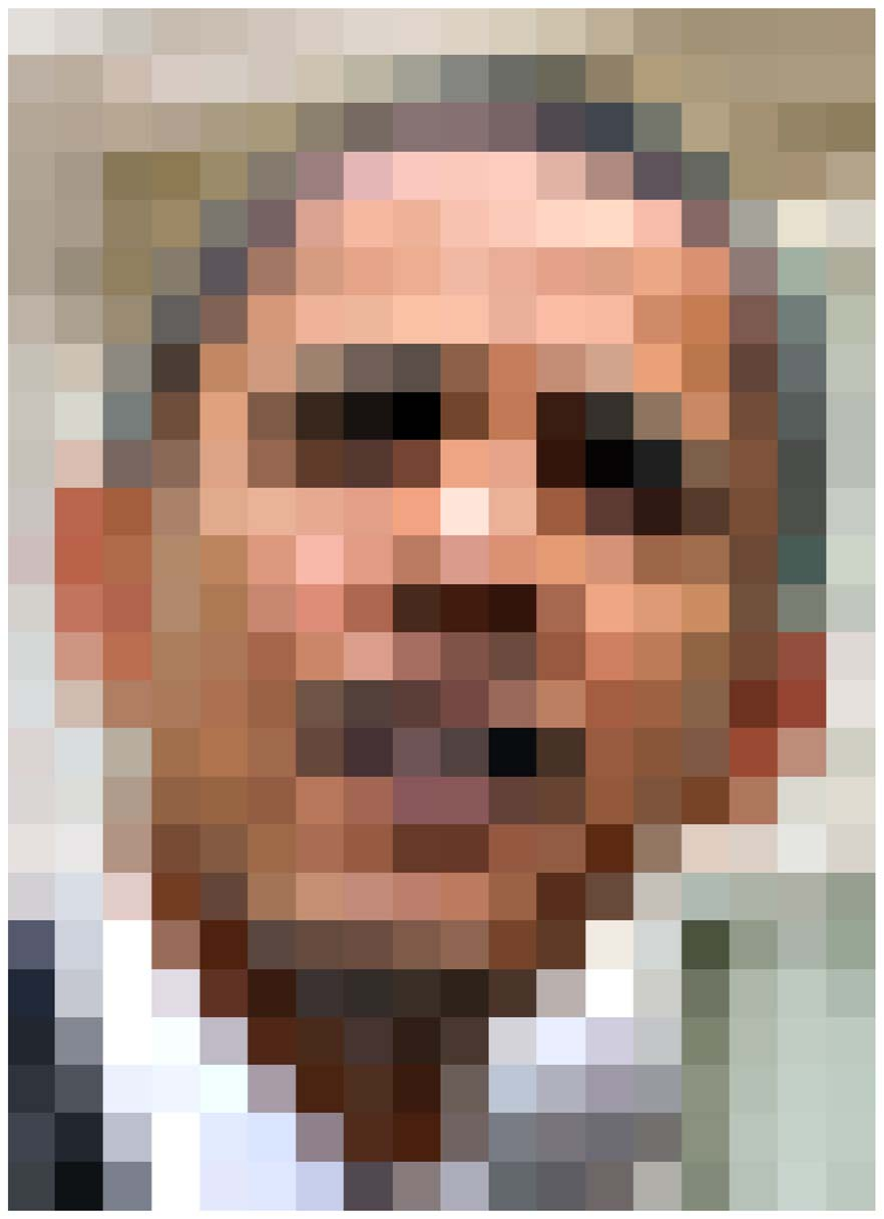
A well-known American. Readers with an interest in current affairs will recognize him from this poor quality image. The face in this image measures 16 pixels wide ×20 pixels high. (Photo credit: Steve Jurvetson).

Previous psychological research has established that humans can identify faces from extremely poor quality images, when they are familiar with the faces concerned [4]–[7]. For example, Yip & Sinha [7] found that viewers could identify blurred photographs of familiar faces with equivalent image resolutions as low as 7×10 pixels (see Figure 1). Here we exploit the robustness of familiar face recognition to mine high-resolution portrait photographs for latent information. Specifically, we show that the faces of hidden bystanders can be identified via reflections in the eyes of photographic subjects. Corneal analysis has previously been used to recover coarse aspects of the physical environmental, such as ambient lighting conditions [8] [9]. The present findings demonstrate that corneal reflections can reveal surprisingly rich information about the social environment too.

Reflection images form readily on the cornea of the eye, potentially revealing features of the subject's surroundings. Indeed, the pupil of the eye derives its name from a reflected onlooker, *pupilla* being Latin for young girl [10]. In practice, the reflection image often extends beyond the pupil and into the iris, capturing regions of the environment that were not visible to the subject when the photograph was taken. Nevertheless, the relative area of such reflection images is small, as an iris typically occupies less than 0.5% of frontal face area. The information that can be extracted from a corneal reflection image is thus limited by the density of elements in the camera's sensor array.

For the current study, we used a 39 megapixel digital camera to take passport-style photographs of volunteer models. In separate exposures, these volunteers served as *subjects*, when they were direct subjects of the photographs, and as *bystanders*, when they were visible only indirectly via the subject's corneal reflection. Pilot work determined that image area for reflected bystander faces was smaller than for subject faces by a factor of around 30,000. The quality of the bystander images is thus poor, despite the high pixel count of the source photographs. To establish whether bystander faces could be identified from the extracted images, we presented them in Experiment 1 as stimuli in a face matching task [11]–[15]. To assess effects of familiarity on match performance, we compared observers who were *familiar* or *unfamiliar* with the faces concerned. In Experiment 2, we assessed spontaneous recognition of the extracted images.

### Ethics Statement

This study was approved by the Ethics Committee of the College of Science and Engineering, University of Glasgow. All participants provided written informed consent and appropriate photographic release.

### Image acquisition

#### High-resolution photography

**Figure 2:**
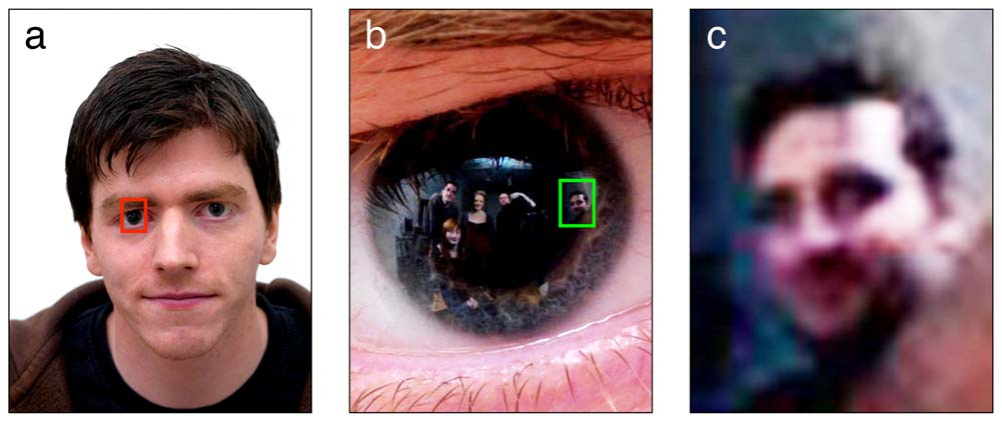
Zooming in on the subject's eye reveals hidden bystanders. (**a**) High-resolution face photograph. The red frame indicates the region of interest, which includes the reflective surface of the cornea. (**b**) Zoomed view of the region of interest with contrast enhanced (see Methods for details of image enhancement). Five bystanders are clearly visible in the corneal reflection. From left to right, RJ (author), CF (seated), IS, SC (photographer), and AS. The green frame highlights the face of bystander AS. (**c**) Enhanced close-up of AS. Gender, ethnicity, hair color, and approximate age can be clearly discerned, along with emotional expression and direction of social attention.

**Figure 3:**
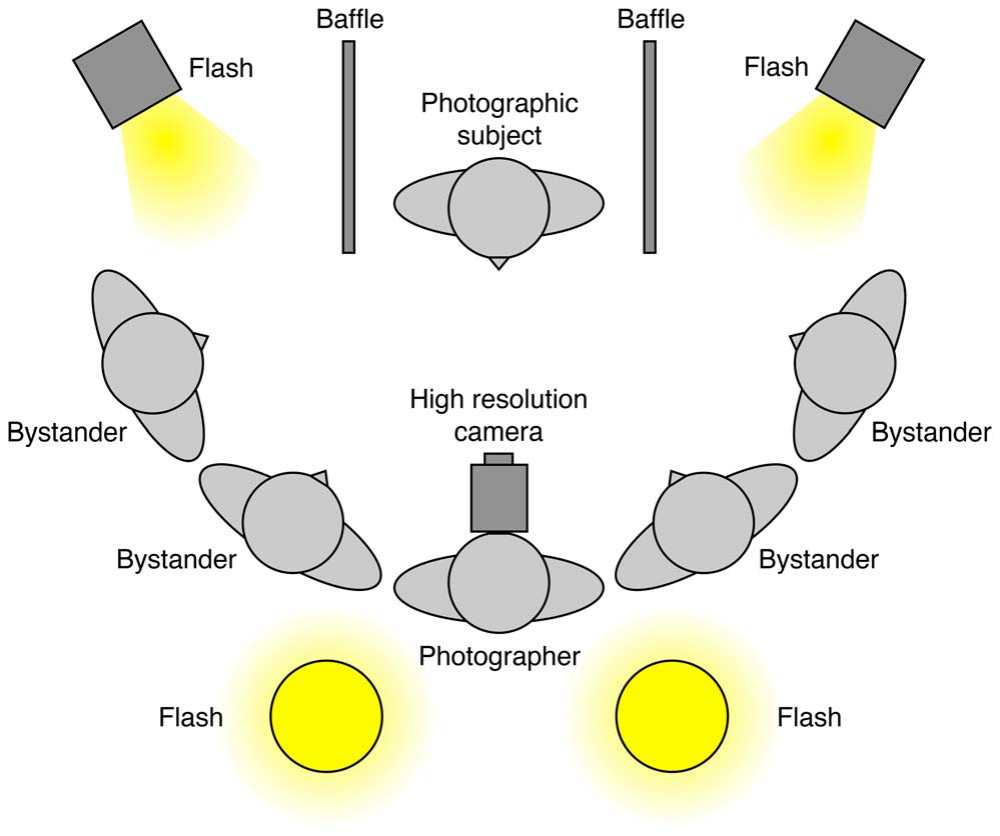
Schematic plan of apparatus and layout for photography. Not to scale.

Eight volunteer photographic subjects (3 female, 5 male; mean age 23.5 years) were processed in two groups of four. Each volunteer thus served as the direct *subject* of one photograph, and as a *bystander* in three other photographs (Figure 2). Subjects were photographed from a viewing distance of approximately 1 m using a Hasselblad H2D 39 megapixel digital camera (50 ISO; f8 aperture; 1/250 sec. shutter; single shot, manual focus) with 120 mm macro lens. The room was flash illuminated by two Bowens DX1000 lamps with dish reflectors, positioned side by side approximately 80 cm behind the camera, and directed upwards to exclude catch light. Two additional DX1000 flash lamps with soft boxes were positioned behind baffles on either side of the subject to illuminate the bystanders. Three volunteer bystanders, plus photographer SC and author RJ stood in an arc formation around the subject at a distance of approximately 1 m (Figure 3). Photographic subjects gave written informed consent, as outlined in the PLOS consent form, to publication of their photographs.

#### Image processing

**Figure 4:**
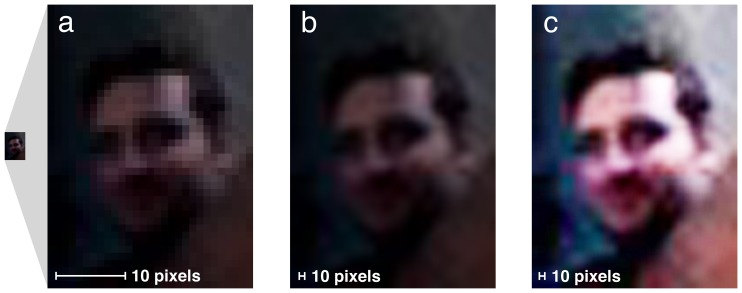
Image processing. (**a**) Example eye reflection extract, magnified to show coarse pixellation in the raw image. (**b**) Resized extract, illustrating the smoothing effect of bicubic interpolation. (**c**) Contrast-adjusted image used for experimental presentation.

The high-resolution photographs measured 5,412 pixels wide by 7,216 pixels high (39,052,922 pixels in total). Whole face area (excluding hair) was approximately 12 million pixels on average, with iris region accounting for approximately 54,000 pixels on average, or less than 0.5% of the whole face area. Each volunteer appeared in the corneal reflection image of three different subjects. From these images, the largest reflection of each bystander's face was selected for presentation in the face matching experiment. Bystander images were extracted as rectangular sections measuring 27 to 36 pixels wide by 42 to 56 pixels high, capturing the head and shoulders of the bystander in roughly passport-style framing (Figure 4). Whole face area for the reflected bystanders was 322 pixels on average, or approximately 0.003% of the whole face area for the photographic subjects. For presentation in the matching experiment, the extracted face images were rescaled to a height of 400 pixels (width 244 to 290 pixels), using bicubic interpolation to reduce high spatial frequency noise. Brightness and contrast were automatically adjusted using the Auto Contrast function in Adobe PhotoShop to improve image definition. Movie S1 shows a continuous zoom from subject to bystander.

## Experiment 1: Face matching

To determine whether the eye reflection images could support identity discrimination, we paired each image with a standard photo of the same face or a similar-looking face in an identity matching task. Observers made ‘same person’ or ‘different person’ judgments for each pair. We predicted that observers would be able to distinguish bystanders from similar-looking foils, especially when they were familiar with the bystanders' faces.

### Method

#### Design and materials

**Figure 5:**
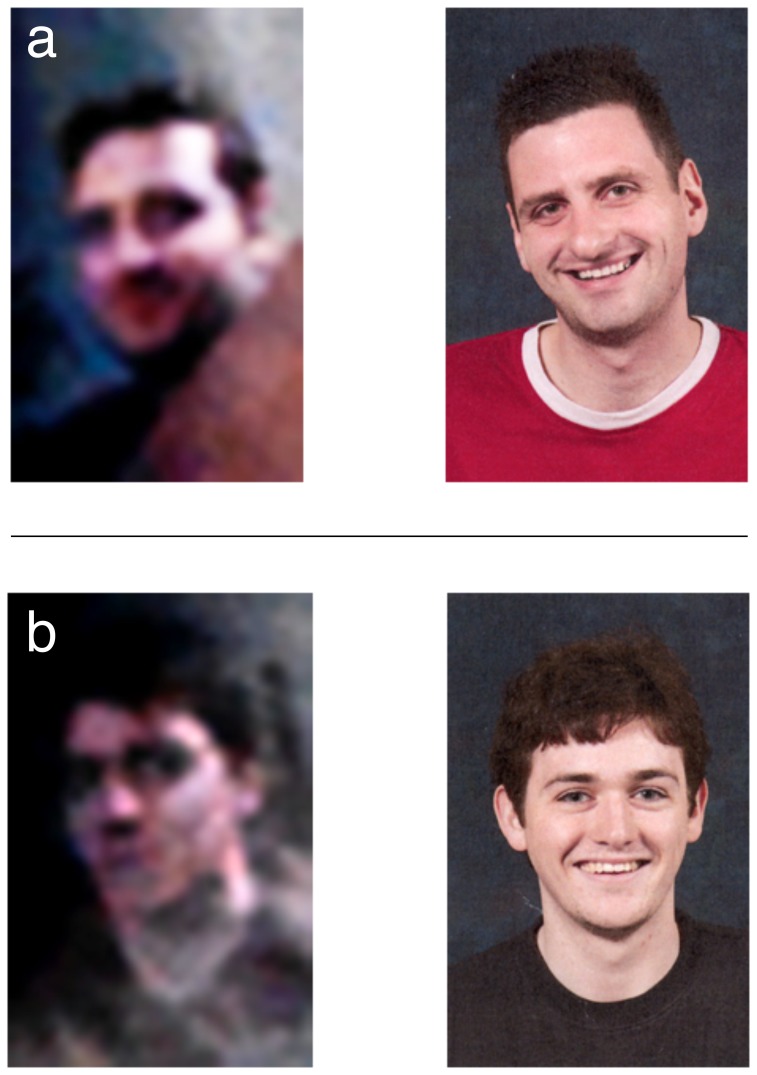
Example pairs from the face matching task in Experiment 1. (**a**) Example *Same Person* pair. (**b**) Example *Different Person* pair. For *Different Person* pairs, basic level descriptors of the foil (e.g. gender, ethnicity, hair color, build, approximate age) matched those of the target, making the task perceptually demanding.

Corneal reflection images for each of the 8 bystanders were paired with comparison photographs of i) the same person (8 *Same Person* pairs) and ii) a different person (8 *Different Person* trials), resulting in 16 image pairs in total. Note that chance performance was 50% in this task. The comparison photos were University enrollment photographs that were taken under homogeneous studio conditions approximately 14 months before the experimental photo session (Figure 5). For *Different Person* pairs, the foil was always the most similar looking person in the cohort of 108 undergraduate students, as determined by three independent raters. Note that each foil met the same general description as target with which it was paired (e.g. young Caucasian male, medium build, short black hair). This ensured that same/different identity judgments required rather subtle perceptual discriminations. The recovered bystander images and comparison photographs were color printed into response booklets at a height of 10.5 cm (width varied between 6.4 cm and 7.6 cm).

#### Participants

Two groups of volunteers took part in the matching task. The Unfamiliar group comprised 16 undergraduate students drawn from non-Psychology departments (10 female, 6 male; mean age 23.3 years). As these Unfamiliar observers were from different departments than the match targets, they were unlikely to have encountered the targets previously. The Familiar group comprised 16 classmates of the targets (14 female, 2 male; mean age 22.5 years). These Familiar observers were drawn from the same final-year undergraduate cohort as the match targets, and were likely to have encountered the target individuals frequently in daily life. Observers' actual familiarity with each face was assessed at the end of the experiment.

#### Procedure

All 16 face pairs (8 *Same Person* pairs; 8 *Different Person* pairs) were presented to participants in a random order. Printed task instructions were provided as follows:

“In this experiment you will be shown pairs of face photographs. In each pair, the photo on the left will be poor quality, and the photo on the right will be good quality. For each pair, your task is to decide whether the two photos show the *same* person or two *different* people. Please indicate your decision by ticking the appropriate box. This is a difficult task. Don't worry if you find it hard - just try your best. There are 16 pairs in total. Please work through these in order, without going back to change your previous answers. There is no time limit for the task. Please take as long as you need for each decision.”

After completing the matching task, participants were presented with an array containing all 16 studio quality comparison photographs (8 targets and 8 foils), and were asked to indicate any individuals whose faces were already familiar to them before the experiment.

### Results

To ensure that the familiarity manipulation was not compromised by items from the opposite category, any faces that were unknown to an observer in the *Familiar* group (<11%) or known to an observer in the *Unfamiliar* group (<2%) were excluded from analysis. Accuracy in the *Unfamiliar* condition was well above chance level of 50%, despite the demanding nature of the matching task [*n* = 16; mean = 71%; s.d. = 11.1; two-tailed *t*(15) = 7.64, *p*<.0001, *d* = 1.91]. Accuracy in the *Familiar* condition exceeded both chance performance [*n* = 16; mean = 84%, s.d. =  2.1, two-tailed *t*(15) = 11.15, *p*<.0001, *d* = 2.79] and performance in the *Unfamiliar* condition [two-tailed *t*(30) = 3.02, *P*<.01, *d* = 1.10], confirming that bystanders' faces could be reliably distinguished from similar foils.

## Experiment 2: Spontaneous recognition

Previous studies have shown that face matching accuracy is a reliable proxy for face recognition accuracy [11]–[13]. Here, we had the opportunity to test recognition directly, by presenting eye reflection images in a face naming task. This experiment was motivated by an anonymous reviewer who reported recognizing author RJ from Figure 2b. To test spontaneous recognition more formally, we presented eye reflection images of RJ and 5 other males in a lineup-style array. Observers who were *familiar* with the face of RJ, and *unfamiliar* with the other faces, were asked to name anyone in the array whom they could identify. We expected that if eye reflection images can be spontaneously recognized, then i) the hit rate (correct naming of RJ) should be high, and ii) the false positive rate (mistaken identification of unknown faces) should be low.

### Method

#### Design and materials

**Figure 6:**
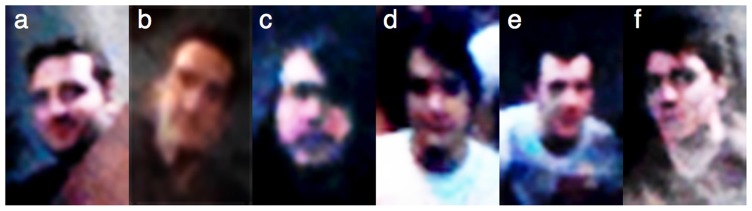
Example face array from the spontaneous recognition task in Experiment 2. (**a**, **c–f**) Corneal reflection images showing bystanders AS, CK, AC, MA, and PD from Experiment 1. (**b**) Corneal reflection image showing author RJ.

Corneal reflection images for each of the 5 male bystanders from Experiment 1, plus author RJ (6 images in total), were used to construct a lineup-style array, which was presented onscreen at 204 pixels high ×745 pixels wide (see Figure 6). Array items were arranged in different random orders for different participants.

#### Participants

Ten new volunteers (2 female, 8 male; mean age 36.9) who were naive to the purpose of the experiment participated. All of these participants were *familiar* with the face of RJ (mean acquaintance 18.2 years), and *unfamiliar* with the faces of the bystanders (zero acquaintance; none had visited the University of Glasgow where the bystanders studied).

#### Procedure

The face array was presented to participants with the following printed task instructions:

“For any face that you can identify, please write in the person's name. Please also indicate your confidence in each decision (i.e. whether or not you know each face) by providing a confidence rating on a scale of 1 to 10 (1 =  guessing, 10 =  completely certain). This is not a trick question, we are just trying to establish how useful images like this might be.”

No time limit was imposed for completing this task.

### Results

Correct naming of the *familiar* face was frequent (hits 90%), and mistaken identification of the *unfamiliar* faces was infrequent (false positives 10%). In addition, confidence ratings were higher for hits (M = 7.89, SD = 1.36) than for false positives (M = 4.80, SD = 3.11), though false positives were too infrequent to allow statistical analysis (n = 5).

## Discussion

By zooming in on high-resolution passport-style photographs, we were able to recover images of bystanders from reflections in the eyes of photographic subjects. Performance in the face matching task (Experiment 1) and the spontaneous recognition task (Experiment 2) indicate that these bystander images were not merely *informative* about facial appearance, they were properly *identifiable* to viewers who knew the faces. This is perhaps a surprising result, given the very unpromising source of these images. However, it is consistent with previous evidence that familiar face recognition is extremely tolerant of poor image quality [16]. We note that the reflection images also contain cues to bystanders' emotional state and interest, via facial expression [17], gaze direction [18], and posture [19], although we did not explore those cues here.

One possible extension of this technique would be to combine pairs of images recovered from the subject's two eyes. In principle, these images contain the stereo disparity information required to reconstruct a 3D representation of the environment from the viewpoint the photographic subject [20]. Since corneal reflections extend beyond the aperture of the pupil, such reconstructions could capture a wider angle of the scene than was visible to the subject at the time (see [21] for a related technique).

For now, our findings suggest a novel application of high-resolution photography: for crimes in which victims are photographed, corneal image analysis could be useful for identifying perpetrators. As with other sources of forensic evidence (e.g. fingerprints), corneal reflection images may not always be readily available. In particular, clear corneal reflections require the subject's face to be in focus, and viewed from a roughly frontal angle under good lighting. They also require high image resolution in order for bystanders' faces to be properly resolved. We note that pixel count per dollar for digital cameras has been doubling approximately every twelve months [22] [23]. This trajectory implies that mobile phones could soon carry >39 megapixel cameras routinely. However, as the current study emphasizes, the extracted face images need not be of high quality in order to be identifiable. For this reason, obtaining optimal viewers - those who are familiar with the faces concerned - may be more important than obtaining optimal images.

## Supporting Information

Movie S1 **Animated zoom on the cornea of a high-resolution photographic subject.** The zoom begins with a passport photo-style framing of the subject, and ends with a full face close-up of a bystander captured in the subject's corneal reflection. Successive movie frames represent a linear magnification of 6%. Each frame was resized to 720 pixels wide ×540 pixels high using bicubic interpolation to reduce high spatial frequency noise. Contrast was enhanced separately for each frame using the Auto Contrast function in Adobe Photoshop to improve definition. The image sequence was then converted to movie format for viewing.
(AVI)
